# Safety and efficacy of co-administered diethylcarbamazine, albendazole and ivermectin during mass drug administration for lymphatic filariasis in Haiti: Results from a two-armed, open-label, cluster-randomized, community study

**DOI:** 10.1371/journal.pntd.0008298

**Published:** 2020-06-08

**Authors:** Christine L. Dubray, Anita D. Sircar, Valery Madsen Beau de Rochars, Joshua Bogus, Abdel N. Direny, Jean Romuald Ernest, Carl R. Fayette, Charles W. Goss, Marisa Hast, Kobie O’Brian, Guy Emmanuel Pavilus, Daniel Frantz Sabin, Ryan E. Wiegand, Gary J. Weil, Jean Frantz Lemoine

**Affiliations:** 1 Centers for Disease Control and Prevention, Atlanta, Georgia, United States of America; 2 University of Florida, Gainsville, Florida, United States of America; 3 Washington University in St. Louis, St. Louis, Missouri, United States of America; 4 RTI International, Washington, District of Columbia, United States of America; 5 IMA World Health, Port-au-Prince, Haiti; 6 Ministère de la Santé et de la Population, Port-au-Prince, Haïti; NIH-NIRT-ICER, INDIA

## Abstract

In Haiti, 22 communes still require mass drug administration (MDA) to eliminate lymphatic filariasis (LF) as a public health problem. Several clinical trials have shown that a single oral dose of ivermectin (IVM), diethylcarbamazine (DEC) and albendazole (ALB) (IDA) is more effective than DEC plus ALB (DA) for clearing W*uchereria bancrofti* microfilariae (Mf). We performed a cluster-randomized community study to compare the safety and efficacy of IDA and DA in an LF-endemic area in northern Haiti. Ten localities were randomized to receive either DA or IDA. Participants were monitored for adverse events (AE), parasite antigenemia, and microfilaremia. Antigen-positive participants were retested one year after MDA to assess treatment efficacy. Fewer participants (11.0%, 321/2917) experienced at least one AE after IDA compared to DA (17.3%, 491/2844, *P*<0.001). Most AEs were mild, and the three most common AEs reported were headaches, dizziness and abdominal pain. Serious AEs developed in three participants who received DA. Baseline prevalence for filarial antigenemia was 8.0% (239/3004) in IDA localities and 11.5% (344/2994) in DA localities (<0.001). Of those with positive antigenemia, 17.6% (42/239) in IDA localities and 20.9% (72/344, *P* = 0.25) in DA localities were microfilaremic. One year after treatment, 84% percent of persons with positive filarial antigen tests at baseline could be retested. Clearance rates for filarial antigenemia were 20.5% (41/200) after IDA versus 25.4% (74/289) after DA (*P* = 0.3). However, 94.4% (34/36) of IDA recipients and 75.9% (44/58) of DA recipients with baseline microfilaremia were Mf negative at the time of retest (*P* = 0.02). Thus, MDA with IDA was at least as well tolerated and significantly more effective for clearing Mf compared to the standard DA regimen in this study. Effective MDA coverage with IDA could accelerate the elimination of LF as a public health problem in the 22 communes that still require MDA in Haiti.

## Author summary

Lymphatic filariasis (LF) also known as “elephantiasis” is caused by infection with filarial worms that are transmitted by mosquitoes. The Global Programme to Eliminate LF (GPELF) aims to interrupt disease transmission through annual rounds of mass drug administration (MDA). Several studies have shown that a single dose of a triple-drug therapy (ivermectin, diethylcarbamazine and albendazole or IDA) was superior to a single dose of the standard double-drug therapy (diethylcarbamazine and albendazole or DA) and might accelerate LF elimination. In Northern Haiti, we performed a large community treatment study to compare the safety and efficacy of three-drug and two-drug therapies. Almost 6,000 participants were treated with IDA or DA and tested for LF. Each participant was assessed for adverse events (AE) and participants who tested positive for LF at enrollment were retested one year after treatment. Less participants who received IDA (11.0%) experienced AEs compared to participants who received DA (17.3%). IDA was also more effective to eliminate microfilariae (adult worm offspring) from the blood compare to DA. Results from this study showed that IDA was well tolerated in Haiti and has the potential to accelerate LF elimination in countries that currently use DA if high MDA coverage can be achieved.

## Introduction

Lymphatic filariasis (LF) is a vector-borne neglected tropical disease caused by the nematode parasites *Wuchereria bancrofti*, *Brugia malayi* and *B*. *timori* [1]. Adult filariae cause severe damage to the lymphatic system leading to chronic, disabling morbidities such as lymphedema, elephantiasis, and hydrocele. The World Health Assembly passed Resolution 50.29 in 1997 that called for elimination of LF as a public health problem [2]. Consequently, the World Health Organization (WHO) launched the Global Programme to Eliminate Lymphatic Filariasis (GPELF) in 2000. To interrupt transmission, WHO recommends community distribution of antifilarial medications to entire at-risk populations through community-based mass drug administration (MDA). Ivermectin (IVM) and albendazole (ALB) are provided as MDA in areas where onchocerciasis is co-endemic with LF; diethylcarbamazine (DEC) and ALB are provided in areas without onchocerciasis or loiasis [3].

The WHO recommends at least five years of annual MDA with effective coverage (with at least 65% of the total population swallowing the medicines) before performing surveys to assess whether MDA can be stopped [4]. In 2018, 52 of the 76 countries endemic for LF still required MDA. Of those, three countries had not started MDA and 11 countries had not implemented MDAs in all endemic districts. Additionally, in some countries that have achieved at least five years of effective MDAs, surveys have indicated that the criteria for stopping MDA have not been met [5]. Therefore, the objective of eliminating LF as a public health problem globally by 2020 will not be achieved using the standard 2-drug MDA regimens.

Several clinical trials have shown that a 3-drug regimen (IVM, DEC and ALB or IDA) is superior to the currently recommended 2-drug regimen (DEC and ALB or DA) for clearing microfilaremia [6–8]. Post-treatment systemic adverse events (AE) were commonly reported after both treatments but were more frequently reported after IDA than after DA. However, no serious AE (SAE) were observed among people taking IDA. The dramatic reduction and sustained clearance of microfilaremia along with the safety profiles seen in these studies suggested that the 3-drug regimen may be a useful tool for accelerating the path towards elimination of LF as a public health problem [9].

Although these studies clearly demonstrated the superiority of IDA for clearing *W*. *bancrofti* microfilariae (Mf) from the blood, additional supportive safety and efficacy data were needed before this triple therapy could be recommended for large scale use as an MDA regimen in LF endemic countries. WHO recommends a best practice called “cohort event monitoring” for demonstrating safety of new drug regimens for public health program use [10]. Cohort event monitoring refers to the use of prospective, observational cohort studies of patients to whom the medicine of interest has been administered. It avoids some of the common deficiencies in drug safety assessment studies such as incomplete reporting, absence of denominators, and investigator biases. The term adverse event refers to an undesirable experience after treatment without regard to whether it is or is not treatment related. A cohort of 10,000 patients is sufficient to provide adequate statistical power to detect serious AEs that occur at a rate equal to or greater than 0.1% [11].

Haiti is one of four countries in the Americas where transmission of LF still occurs [5]. In 2001, a national survey undertaken by the Ministère de la Santé Publique et de la Population (MSPP) and partners indicated that approximately 90% of Haiti’s 140 communes (equivalent to districts) required MDA; this corresponded to an at-risk population of nearly 8 million people. Haiti follows the WHO’s LF elimination strategy by providing annual MDA with DEC and ALB for a minimum of five years. Even though LF was not identified in every commune, all communes were targeted for MDA. MDA was provided in one commune in 2000, and MSSP achieved full geographic coverage with MDA in 2012 [12, 13]. By 2019, 87% (122/140) of communes in Haiti no longer required MDA. Despite these successes, a persistent challenge has been continued LF transmission, even after 10 years of MDA, in several communes that had high baseline LF prevalence (10−45% antigenemia) [14]. The increased efficacy of IDA represents an opportunity to address this challenge.

To meet the WHO requirement of establishing the safety of IDA MDA in at least 10,000 people, community safety studies were conducted with IDA and DA for cohort event monitoring in five countries (Haiti, Indonesia, India, Fiji and Papua New Guinea) with different LF species and mosquito vectors [15]. Here we report detailed results from the Haiti study. The primary objective was to compare the frequency, type and severity of AEs following MDA with IDA versus DA in infected and uninfected individuals. A secondary objective of the study was to compare the efficacy of the two regimens for clearing microfilaremia and filarial antigenemia as assessed one year after treatment.

## Methods

### Ethics statement

Ethical approval for the study was granted by the National Bioethics Committee (1718–11) of the Ministry of Public Health and Population (MSPP) of Haiti. All participants aged 18 years and older provided written informed consent before any study procedures were done. Participation of children (7−17 years of age) required their written assent and the written permission of one parent or guardian; participation of children 5–6 years of age only required permission from one parent or guardian. If a participant was unable to read or write, another literate person who was not involved in the study (family member, neighbor or other community member) could act as a witness to the consenting process. A copy of the information sheet and the signed consent/assent form with a phone contact number was given to each participant.

### Study setting

**Fig 1:**
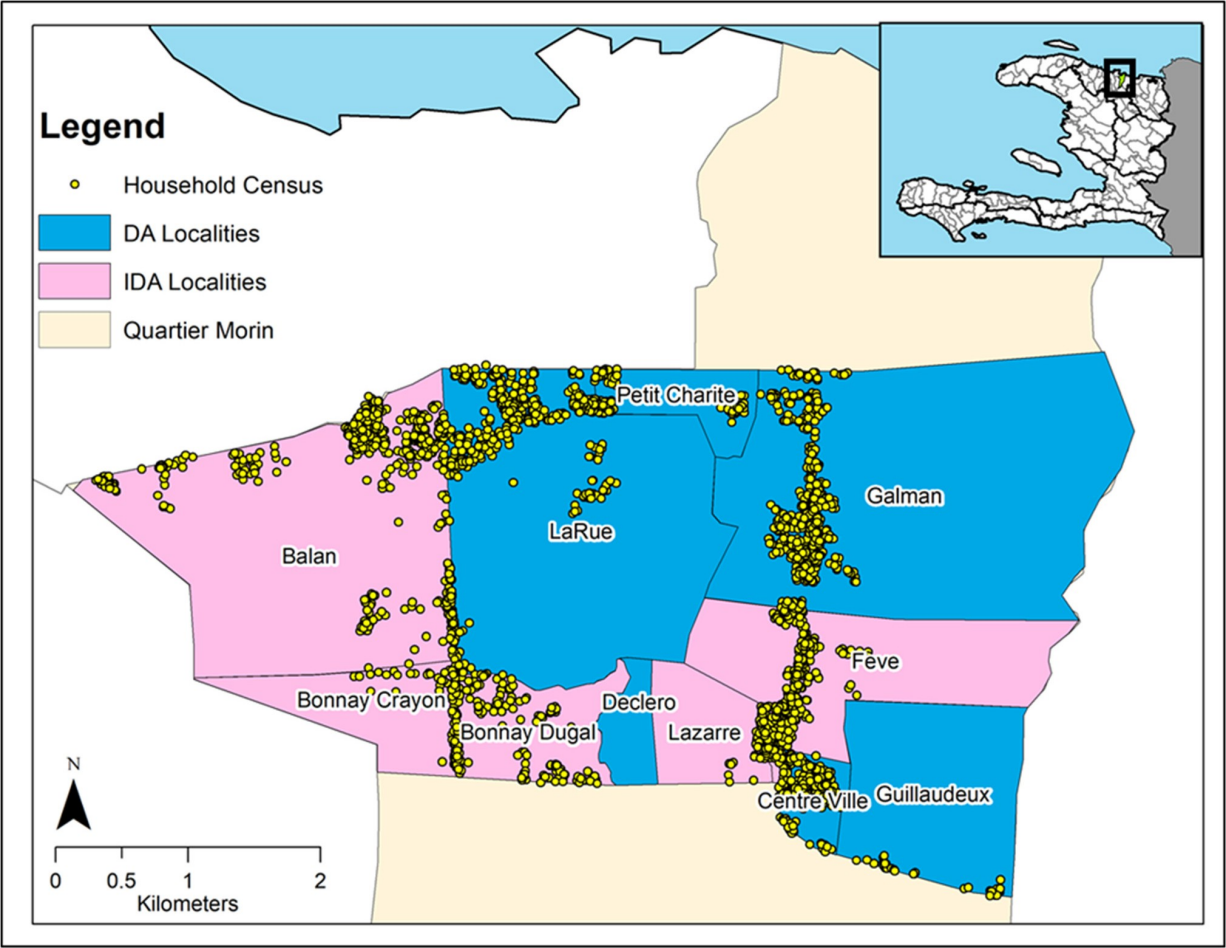
Localities randomly assigned to treatment regimen (DA or IDA) and position of houses included in the 2016 census. DA, diethylcarbamazine plus albendazole; IDA, ivermectin plus diethylcarbamazine and albendazole.

The study was conducted between October 31, 2016 and February 10, 2017 in 10 localities of the Commune of Quartier Morin (19.6967 N, -72.1586 W) located in the Northern Department of Haiti (Fig 1). A “commune” is the third-level geographic division in Haiti (equivalent to a district in many other countries), and a “locality” is equivalent to a sub-district. The baseline antigenemia prevalence in the commune of Quartier Morin was high, estimated at 39% in 2001. WHO recommends that to stop MDA, microfilaremia or antigenemia prevalence should be under pre-determined thresholds of <1% or <2%, respectively. A survey among residents > 5 years of age performed in 2014 after seven consecutive annual rounds of MDA found that the prevalence of LF was still too high to stop MDA in the Commune of Quartier Morin. The filarial antigenemia prevalence was 4.2% (21/496) and 38% of those with antigenemia were Mf positive (eight positives, 1.6% of all persons tested). Some of the localities included in the current study are communities that were surveyed in 2014. In July 2016, in preparation for the present study, a census enumerated a total of 12,383 people living in 2,669 households in the 10 localities of the Quartier Morin commune selected for the study (Fig 1). Females comprised 53.4% of residents, and 8.2% of residents were younger than five years of age.

### Study design and enrollment of participants

We used an open-label, cluster-randomized community study design to study the relative safety and efficacy of IDA vs. DA in the 10 localities mentioned above. Each locality was randomly assigned to receive either the IDA regimen consisting of a single dose of IVM (200 μg/kg) + DEC (6 mg/kg) + ALB (400 mg) (5 communities) or the DA regimen consisting of a single dose of DEC (6 mg/kg) +ALB (400 mg) (5 communities) (Fig 1). All eligible residents of the 10 localities were invited to participate in the study. Eligible residents were ≥5 years old, were able to provide consent (or assent plus parental consent for minors), had no history of allergy to the study medications, and were not pregnant or suffering from severe chronic illnesses. Women who reported that their last menstrual period started ≥4 weeks before enrollment or who did not recall the date of their last menstrual period were excluded. The core study team, assisted by community health workers who had participated in prior rounds of MDA, visited each house included in the July 2016 census to enrol participants.

### Procedures

After consenting and prior to evaluation for LF infection and treatment, participants were enrolled and assigned a unique identification number. Four trained enrollment teams weighed and measured the height of each participant and recorded their age, sex, general health status and (for females) date of last menstrual period. Then, the enrollment nurse asked each participant about symptoms that corresponded to each of the AEs that were to be assessed after the treatment to establish a baseline.

Laboratory technicians tested each participant for circulating filarial antigenemia (CFA) using the Alere Filariasis Test Strip (FTS, Alere) performed according to the manufacturer’s instructions. Capillary blood (75μl) was placed on the sample application pad and the result was read at 10 minutes. Since the intensity of antigen test lines is correlated with CFA levels, the results were scored as previously described [16, 17]: 0 for tests with no visible test (T) line; 1 when the T line was weaker than the control (C) line; 2 when the T line was approximately as dark as the C line; and 3 when the T line was darker than the C line. FTS positive participants were tested for microfilaremia at night (from 10 pm−12 am) by thick blood smear examination. Three parallel lines of blood (20 μl each) were placed on slides. Dried slides were stained with Giemsa and examined by trained microscopists.

A dosing table was used to determine the number of IVM (200 μg /kg) and DEC (6mg/kg) tablets for each participant based on their weight. ALB was provided at a fixed dose of 400 mg. Treatment was directly observed to ensure that all enrolled individuals swallowed the tablets. Doses vomited shortly after administration were replaced. If the FTS was positive, the treatment was administered at night after blood was collected for Mf testing.

### Safety study

Adverse events (AE) were defined as any unfavorable and unintended abnormal laboratory finding, symptom or disease that occurred during the study, regardless of whether it was related to the intervention. AEs were classified as mild (grade 1), moderate (grade 2), severe (grade 3), life-threatening (grade 4) and death (grade 5) according to a modified grading system based on the Common Terminology Criteria for Adverse Events (CTCAE) from National Cancer Institute [18]. Serious AEs (SAEs) were defined as any AE that resulted in any of the following outcomes: death, life-threatening event (immediate risk of death), hospitalization or prolongation of existing hospitalization, persistent or significant disability or incapacity, or congenital anomaly/birth defect. In addition, other important medical events could be judged to be a SAE if, based upon appropriate medical judgment, the event was believed to jeopardize the participant or require medical or surgical intervention to prevent one of the outcomes listed above. The relationship between each reported or observed SAE and the study medications was investigated and judged by a medical officer to be definitely, probably, possibly, unlikely, or unrelated to treatment. Nurses and physicians collected AE information in an electronic case report form (eCRF). An initial period of active monitoring required a daily house visit by a nurse during the two days following treatment (day 1−2 of follow up). Participants who developed AEs during the next five days (day 3−7, passive follow up) were asked to either contact a study nurse who was in their area or call a hotline monitored by a study staff member and available 24 hours a day and seven days a week. Any AE greater than grade 2 was evaluated by a medical officer who had received specific training for the study. The medical officer on duty completed an AE Evaluation and Report Form for any grade 3, 4 or 5 AE and for all participants who required overnight hospitalization. The physician provided any required immediate treatment and facilitated admission into a local hospital as deemed appropriate. Participants with AEs greater than grade 2 were seen daily by a physician or a nurse until their symptoms resolved. Assessment and management of AEs was free of charge for all participants.

### Efficacy study

The objective of the efficacy study was to assess responses to treatment and to compare the efficacy of the two treatment regimens for clearing microfilaremia and CFA. Therefore, participants who were positive for CFA (FTS) at baseline were retested one year after treatment for CFA and microfilaremia as described above.

### Data acquisition, transfer, and management

An electronic data capture (EDC) system developed by CliniOps (Fremont, CA, USA) was used to compile the data into two databases: baseline and efficacy. Deidentified data were entered directly into a tablet via a mobile data management application called CliniTrial. The data were entered by a designated trained member of each survey team on the day of enrollment, AE assessment, or at the 1-year follow up. The EDC system is 21 CFR Part 11 compliant. eCRFs were developed to comply with International Council for Harmonization on Good Clinical Practice (ICH GCP) and Clinical Data Acquisition Standards Harmonization / Clinical Data Interchange Standards Consortium (CDASH/CDISC) standards [19]. User acceptability testing was performed for eCRFs prior to their deployment. Validation checks and automated alert checks were programmed into the EDC system to maintain a high level of data quality at point of entry. Data were synchronized regularly through a secured server. AEs were coded using MedDRA dictionaries (version 20.0) [20]. Paper CRFs were used for backup in case of EDC or equipment malfunction and for documentation of SAEs. All written forms (i.e., consent and backup data collection forms) were stored at the endemic country collaborator’s institution as per in-country institutional review board (IRB) requirements for storage of source documents.

### Sample size and statistical analysis

The WHO requires a total of 10,000 participants to detect with high confidence a SAE rate of less than 0.1%. In Haiti, we aimed to enroll 3,000 participants per drug regimen to make a substantial contribution to the global, multi-site sample size, which was reached by including participants in Haiti and in the four other countries.

Data were analyzed with SAS version 9.4 (SAS Institute Inc., Cary, NC, USA). Baseline characteristics of the cohort including CFA and Mf positivity were compared between study arms using chi-square tests and Wilcoxon rank sum tests as appropriate. At baseline, the proportion of FTS and Mf positivity was compared by sex, age, and locality using chi-square tests.

The primary objective for the safety study was to document the rate of AEs that occurred among participants within the first seven days post treatment (day 1−7) by treatment group. This was calculated by dividing the number of participants with at least one AE by the number of participants assessed. The AE outcome (measured at the individual level) was an AE of any severity during the 7-day follow up period. We analyzed this dichotomous outcome using a generalized linear mixed model (GLMM) that assumed a binomial distribution and a logit link function. Locality was treated as a random effect to account for correlation among participants within localities. The association between drug treatment (IDA vs DA) and AEs was assessed using both a univariable logit model and a multivariable logit model where we adjusted the treatment effect for the following covariates: age group (< 18 years and ≥ 18 years), sex, and Mf and CFA positivity.

The objective of the efficacy study was to compare changes among CFA positive participants at baseline in CFA and Mf prevalence one year after treatment both overall and by study regimen using chi squared tests. Among CFA positives, the proportions of participants in each FTS score category (1–3) and the proportions of participants Mf positive participants were compared between baseline and 1-year follow-up using chi squared tests. Among Mf positives, the geometric mean Mf counts per 1 mL of blood were compared at baseline and at follow up using a test on log-transformed values. Among CFA and Mf positive participants at baseline, we compared the rate of conversion to negative status at 12-month follow-up by treatment regimen. *P*-values < 0.05 were considered statistically significant.

## Results

### Enrollment and baseline results

**Fig 2:**
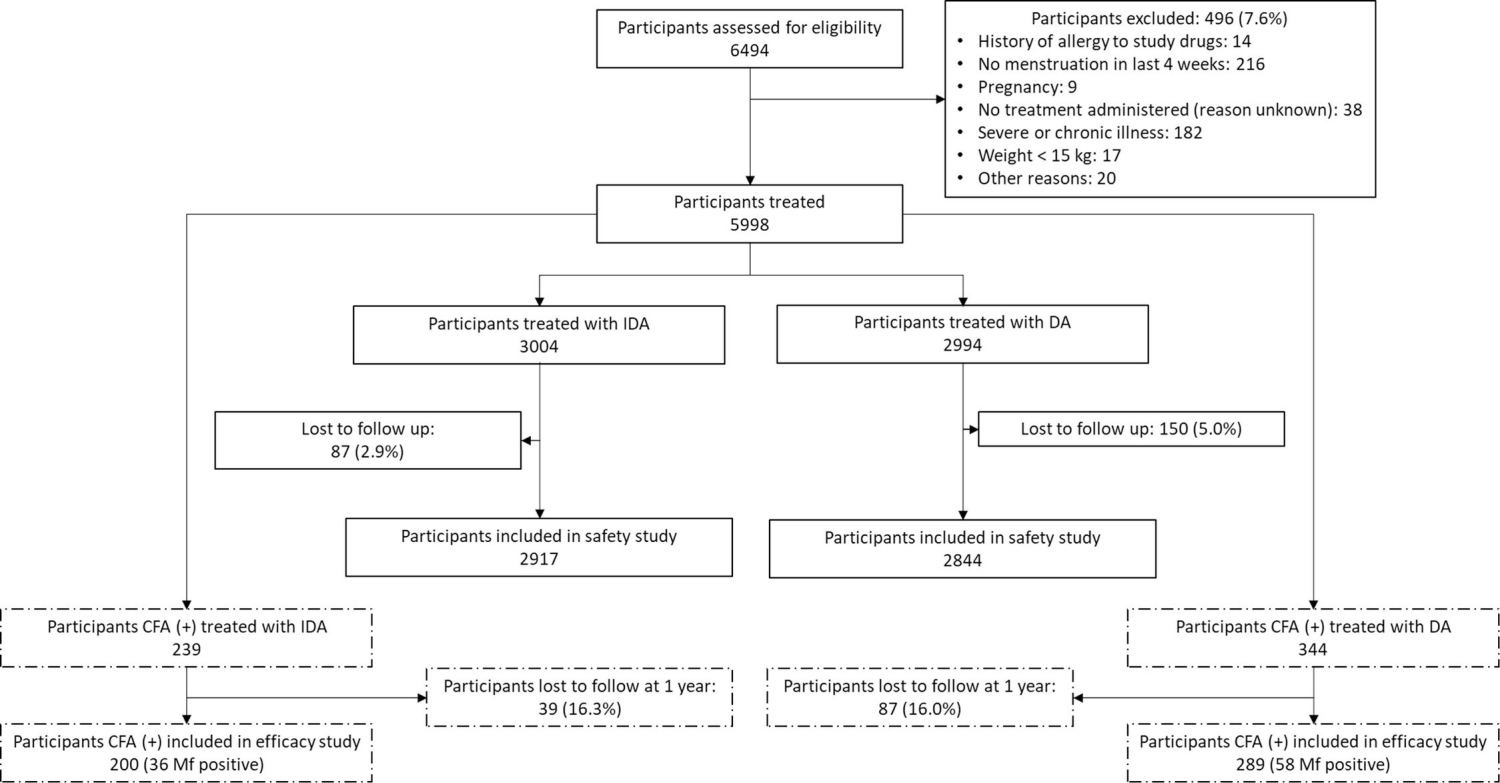
**Study diagram for participants included in the safety (solid line) and efficacy study (dash line).** DA, diethylcarbamazine plus albendazole; IDA, ivermectin plus diethylcarbamazine and albendazole; CFA, circulating filarial antigenemia; Mf, microfilariae. Cluster randomization was used for treatment allocation for each locality.

In the 10 localities, 6,494 people agreed to participate in the study, which represented 52.4% (6,494/12,383) of the census population. Of these, 5,998 (92.4%) were eligible and included in the safety study. Based on random assignment of localities to drug regimens, 3,004 participants from five localities received IDA and 2994 participants from the five remaining localities received DA (Fig 2). Thus 48.4% (5,998/12,383) of the population enumerated in the census received treatment: 47.3% (3,004/6,355) in the localities receiving IDA and 49.7% (2,994/6,028) in the localities receiving DA (Fig 2). The inclusion rates by locality ranged from 39.5% to 64.9%. Because the arrival of ivermectin tablets in Haiti was delayed, the study teams first enrolled people living in the localities assigned to DA.

Baseline demographic and filarial infection data are provided in Table 1. The median age of participants was 18 years in the IDA and DA groups (*P* = 0.2). Similar proportions of female participants were included in the DA and IDA groups (52.8% vs 53.5%, *P* = 0.5). The CFA prevalence was 9.7% (583/5,993, 95% confidence interval [CI] = 9.0–10.5%) and the Mf prevalence was 1.9% (114/5,987, 95% CI = 1.6–2.3%). The geometric mean Mf count for those with microfilaremia was 250/mL (range 17–8,567). More participants included in the DA treatment area were antigen-positive compared to the DA area (11.5% vs. 8.0%, *P* < 0.01). Similarly, more participants in the DA treatment area were Mf positive compared to the DA area (2.4% vs. 1.4%, *P* = 0.004). Most of the CFA positive participants in both treatment areas had strongly positive FTS results (scores of 3) at baseline (Table 1). Mf prevalence at baseline in persons with FTS scores of 1, 2, or 3 were 2.8%, 6.7%, and 38.1%, respectively.

**Table 1:** Baseline demographic characteristics and infection status of participants by treatment area at enrollment, October 2016−February 2017.

	Total n (%)	IDA n (%)	DA n (%)	P value*
**Demographics**				
Female	3,189 (53.2)	1,609 (53.5)	1,580 (52.8)	0.5
Male	2,809 (46.8)	1,395 (45.4)	1,414 (47.2)	-
**Age**				
median (range)^1^	18 (5–97)	18 (5–95)	18 (5–97)	0.2
5–9 years	1,200 (20.0)	592 (19.7)	608 (20.3)	0.04
10–19 years	2,032 (33.9)	1,067 (35.5)	965 (32.2)	-
20–29 years	1,217 (20.3)	601 (20.0)	616 (20.6)	-
30–39 years	727 (12.1)	354 (11.8)	373 (12.5)	-
40–49 years	407 (6.8)	207 (6.9)	200 (6.7)	-
50+ years	415 (6.9)	183 (6.1)	232 (7.8)	-
**Infection Status**				
CFA positive^2^	583 (9.7)	239 (8.0)	344 (11.5)	<0.001
FTS Score 1^#^	145 (24.9)	47 (19.7)	98 (28.5)	0.05
FTS Score 2^#^	180 (30.9)	80 (33.5)	100 (29.1)	-
FTS Score 3^#^	258 (44.2)	112 (46.8)	146 (42.4)	-
Mf positive^3^	114 (1.9)	42 (1.4)	72 (2.4)	0.004
Mf geometric mean^1^ (Mf /ml)	250	305	217	0.2
Mf range (Mf /ml)	17–8,567	17–5,667	17–8,567	-
**Total (N)**	5,998	3,004	2,994	

*Chi square P value unless otherwise noted

^1^P value is from T test on transformed values

^2^N is 5993

^3^N is 5987

^#^ Percent represent the proportion of positive FTS test by score divided by the total number of positive tests

DA, diethylcarbamazine plus albendazole; IDA, ivermectin plus diethylcarbamazine and albendazole; CFA, circulating filarial antigenemia; Mf, microfilariae; FTS, Filariasis Test Strip (Alere)

**Fig 3:**
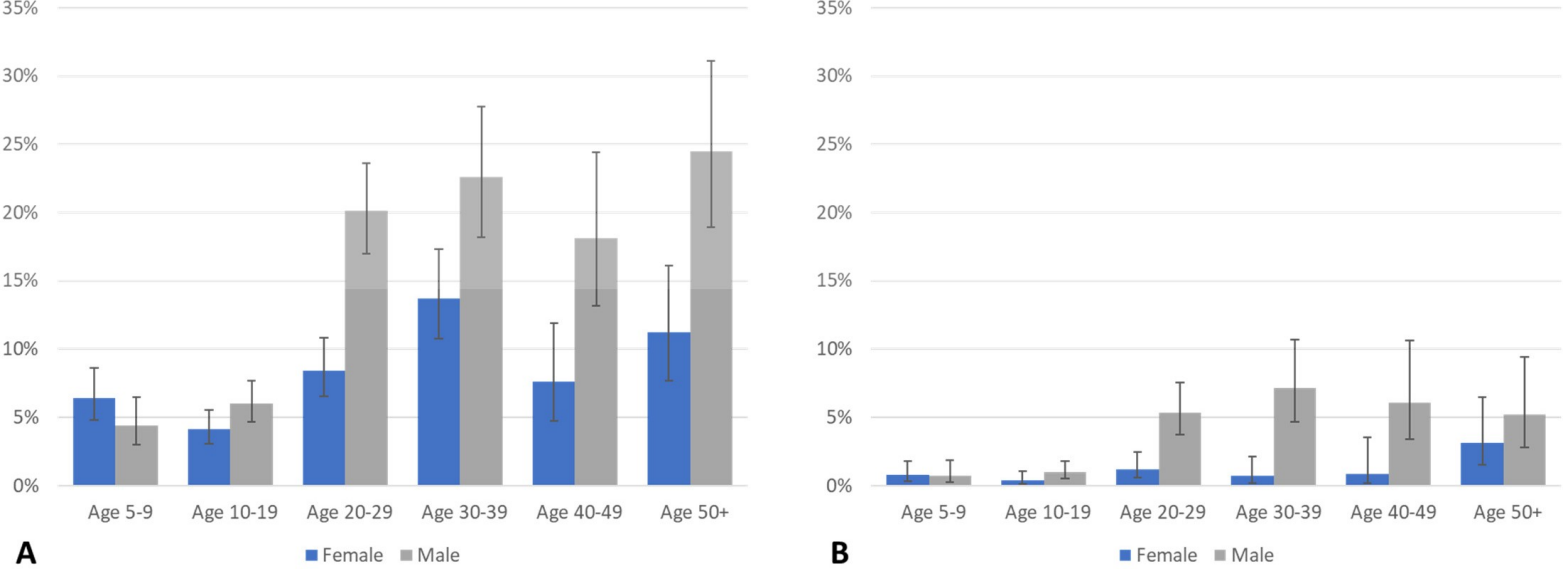
**Proportion of participants CFA positive (A) and Mf positive (B) by age-group and sex before receiving treatment (baseline).** CFA, circulating filarial antigenemia; Mf, microfilariae.

**Fig 4:**
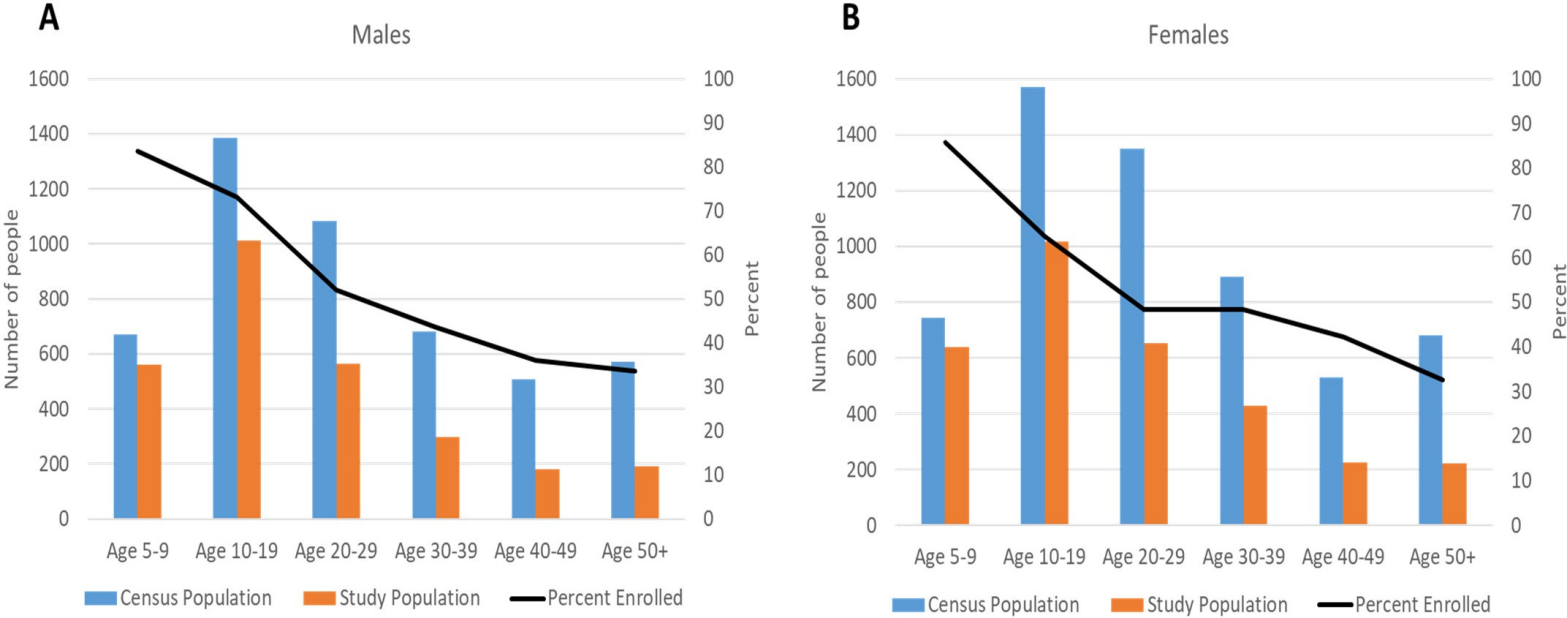
Males (A) and females (B) included in the census who enrolled in the study by age-group.

Before treatment, more men than women were CFA and Mf positive in both treatment groups, and prevalence by Mf and CFA was significantly higher in older participants (Table 2 and Fig 3). Compared to the July 2016 census, the enrolled study population had a similar sex distribution (46.8% male in study compared to 45.9% male in census); however, older age groups (who were more likely to be CFA or Mf positive) were under-sampled among both sexes (Fig 4). When adjusted for age and gender by population weights, the CFA prevalence was 11.1% overall (95% CI = 10.2–11.9%), 14.6% in males (95% CI = 13.2–16.1%) and 8.0% in females (95% CI = 7.0–9.0%). Similarly, the age corrected Mf prevalence at baseline was 2.3% overall (95% CI = 1.9–2.7%), 3.8% in males (95% CI = 3.0–4.6%) and 1.0% in females (95% CI = 0.6–1.4%). The infection rate also varied by locality (Table 2). The proportion of participants with positive FTS results ranged from 4.6% (53/1,148, 95% CI: 3.4%–5.8%) in Balan to 16.0% (16/100, 95% CI: 8.8%23.2%) in Lazarre; the proportion of participants with microfilaremia ranged from 0.4% (5/1,148, 95% CI: 0.1%-0.8%) in Balan to 3.8% (41/1,082, 95% CI: 2.8%-5.1%) in Galman.

**Table 2:** Baseline characteristic by infection status, October 2016−February 2017.

	CFA positive		Mf Positive		Total
	n (%)	P value*	n (%)	P value*	
**Total**	583 (9.7)		114 (1.9)		5993**
**Sex**					
Female	237 (7.4)	<0.001	28 (0.9)	<0.001	3,187
Male	346 (12.3)	-	86 (3.1)	-	2,806
**Age in years**					
Age 5–9	64 (5.3)	<0.001	8 (0.7)	<0.001	1,200
Age 10–19	103 (5.1)	-	14 (0.7)	-	2,032
Age 20–29	168 (13.8)	-	38 (3.1)	-	1,215
Age 30–39	126 (17.4)	-	24 (3.3)	-	726
Age 40–49	50 (12.3)	-	13 (3.2)	-	406
Age 50+	72 (17.4)	-	17 (4.1)	-	414
**Locality (treatment)**					
Balan (IDA)	53 (4.6)	<0.001	5 (0.4)	<0.001^	1,148
Bonnay Dugal (IDA)	27 (7.6)	-	6 (1.7)	-	357
Bonnay Crayon (IDA)	31 (10.0)	-	4 (1.3)	-	310
Feve (IDA)	112 (10.3)	-	24 (2.2)	-	1,088
Lazarre (IDA)	16 (16.0)	-	3 (3.0)	-	100
Petite Charite (DA)	15 (6.9)	-	1 (0.5)	-	217
La Rue (DA)	59 (7.2)	-	4 (0.5)	-	821
Guillaudeaux (DA)	12 (8.8)	-	4 (2.9)	-	137
Centre Ville (DA)	100 (13.6)	-	22 (3.0)	-	733
Galman (DA)	158 (14.6)	-	41 (3.8)	-	1,082

*Chi square P value unless otherwise noted; ^Monte Carlo estimate for Fisher’s exact P value

** N is 5987 for participants tested for Mf

CFA, circulating filarial antigenemia; Mf, microfilaremia; DA, diethylcarbamazine plus albendazole; IDA, ivermectin plus diethylcarbamazine and albendazole

### Safety study results

For the safety study, 96.0% (5761/5998) of participants treated were visited at least once during the 7-day follow-up period (2917 after IDA and 2844 after DA). During the active follow up, 90.5% (5,430/5,998) of participants were visited during the first day of follow up and 88.5% (5,311/5998) during the second day; 96.0% (5,758/5,998) were visited on either day and 83.1% (4,983/5,998) were visited both days. Overall, 14.1% (812/5761) of participants that had AEs assessed reported at least one AE during the week following treatment. The intracluster correlation coefficient for AE was low (0.02). Unexpectedly, more participants receiving DA (17.3%, 491/2,844) reported an AE compared to participants who received IDA (11.0%, 321/2917) (Table 3). Most AEs reported were mild, 88.7% (436/491) of all AEs in the DA arm and 93.4%, (300/321) of all AEs in the IDA arm (Table 3). More women reported AEs than men in both treatment areas (Table 3). Finally, people ≥ 18 years old reported more AEs (17.9%, 525/2927) compared to participants <18 years old (10.1%, 287/2834).

**Table 3:** Frequency of AEs and maximum AE grade/subject after treatment by type of treatment and gender, October 2016−February 2017.

Drug regimen	Gender	# treated n	Any AE n (%)	Mild n (%)	Moderate n (%)	Severe n (%)	SAE n (%)
DA	Female	1529	324 (21.2)	289 (18.9)	27 (1.8)	8 (0.5)	0
	Male	1315	167 (12.7)	147 (11.2)	16 (1.2)	1 (0.1)	3 (0.2)
	Total	2844	491 (17.3)	436 (15.3)	43 (1.5)	9 (0.3)	3 (0.1)
IDA	Female	1568	207 (13.2)	197 (12.6)	7 (0.4)	3 (0.2)	0
	Male	1349	114 (8.5)	103 (7.6)	10 (0.7)	1 (0.1)	0
	Total	2917	321 (11.0)	300 (10.3)	17 (0.6)	4 (0.1)	0

DA, diethylcarbamazine plus albendazole; IDA, ivermectin plus diethylcarbamazine and albendazole; AE, adverse event; SAE, serious adverse event

No participant experienced a SAE after IDA (0%, 0/2917, 95% CI 0.0–0.1)). Three participants (0.1%, 3/2,844, 95% CI 0.0–0.3) experienced a SAE after DA (Table 3). The first person with a SAE was a 78 years old male (CFA positive) who was hospitalized for evaluation of hypertension, urinary tract infection and anemia. The second SAE was in a 35 years old male (CFA and Mf positive) who was hospitalized for evaluation of dysuria, nausea, vomiting and fever. The third person with a SAE was a 13 years old male with acute lower abdominal pain. His evaluation revealed ascariasis, *H*. *pylori* infection. Though AEs in participants with SAEs were graded no higher than three, these AEs were classified as serious, because participants were admitted overnight in the hospital ward for evaluation and observation for at least 24 hours. All three SAEs resolved within 48 hours. Two of the SAEs were considered by attending physicians to have been possibly related to treatment, and one (the 13 years old male) was considered to have been probably related to treatment. A more extensive clinical summary of these SAEs has been published elsewhere [15].

AEs were more frequent following treatment in Mf positive participants but AE rates in persons with microfilaremia were similar after IDA treatment (34.1%, 14/41) and after DA treatment (39.4%, 26/66) (Table 4). For persons with microfilaremia, pre-treatment Mf counts were significantly higher in persons who experienced AEs after treatment compared to persons who did not experience AEs (geometric means: 20.98 Mf/mL vs. 8.81 Mf/mL, *P* = 0.002).

**Table 4:** Frequency of AEs and maximum AE grade/subject after treatment by type of treatment and Mf status, October 2016–February 2017.

Drug regimen	MF test results	# treated n	Any AE n (%)	Mild n (%)	Moderate n (%)	Severe n (%)	SAE n (%)
DA	Mf (-)	2769	464 (16.8)	415 (15.0)	39 (1.4)	8 (0.3)	2 (0.1)
	Mf (+)	66	26 (39.4)	21 (31.8)	3 (4.5)	1 (1.5)	1 (1.5)
	Total	2835	490 (17.3)	436 (15.4)	42 (1.5)	9 (0.3)	3 (0.1)
IDA	Mf (-)	2875	307 (10.7)	288 (10.0)	15 (0.5)	4 (0.1)	0
	Mf (+)	41	14 (34.1)	12 (29.3)	2 (4.9)	0	0
	Total	2916	321 (11.0)	300 (10.3)	17 (0.6)	4 (0.1)	0

11 subjects with missing values for Mf and 10 subjects with missing values for Mf and AE

DA, diethylcarbamazine plus albendazole; IDA, ivermectin plus diethylcarbamazine and albendazole; Mf, microfilariae; AE, adverse event; SAE, serious adverse event

**Fig 5:**
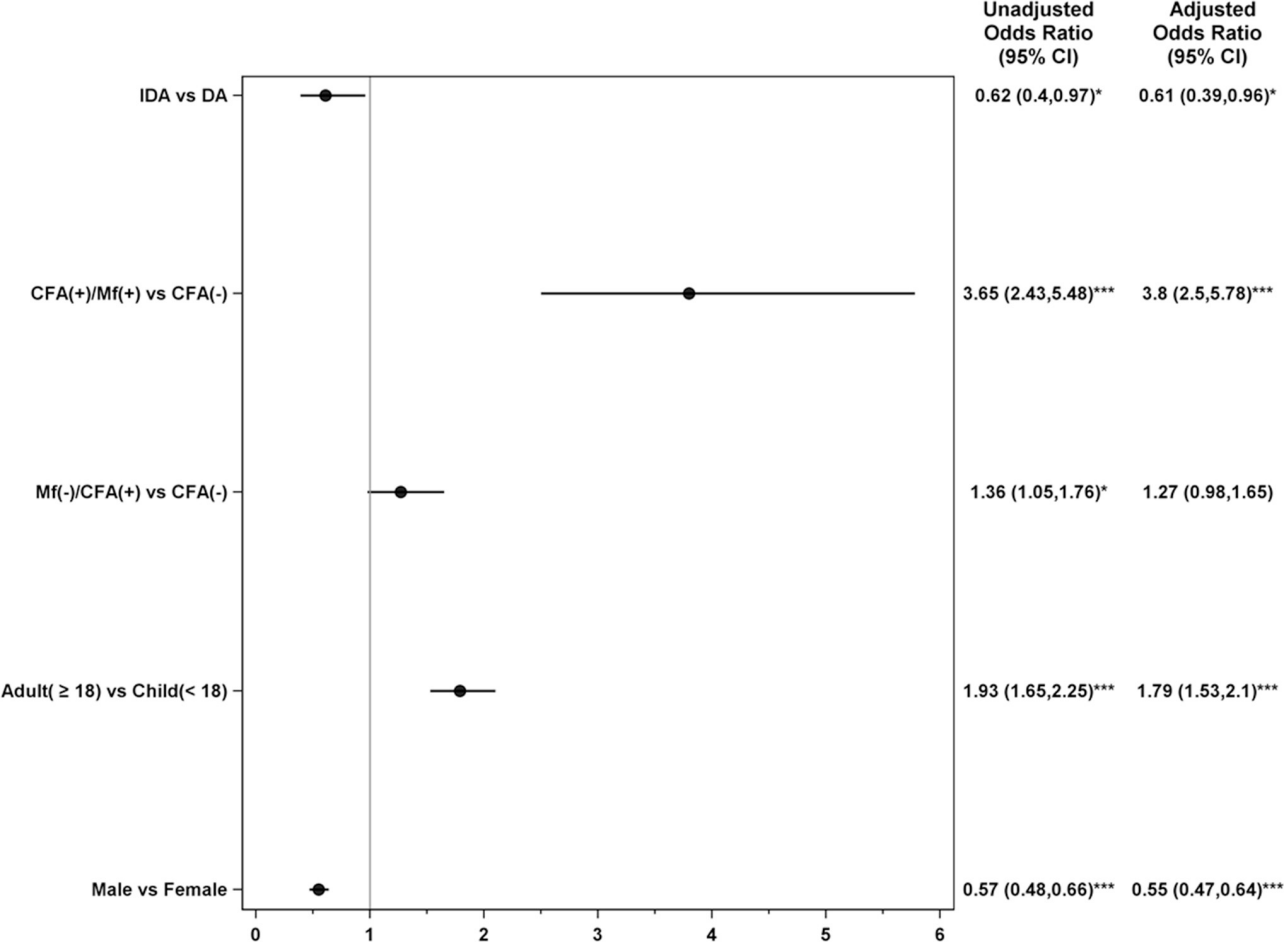
Forest plot showing adjusted odds ratios for factors associated with adverse events following treatment for lymphatic filariasis.

A multivariable logistic regression analysis showed that after controlling for age, sex and infection status, the risk for experiencing an AE was significantly lower for a participant who received IDA compared to a participant who received DA. Microfilaremia, isolated antigenemia (amicrofilaremic), age >18 years, and female sex were also significantly associated with AEs in the multivariable model (Fig 5).

**Fig 6:**
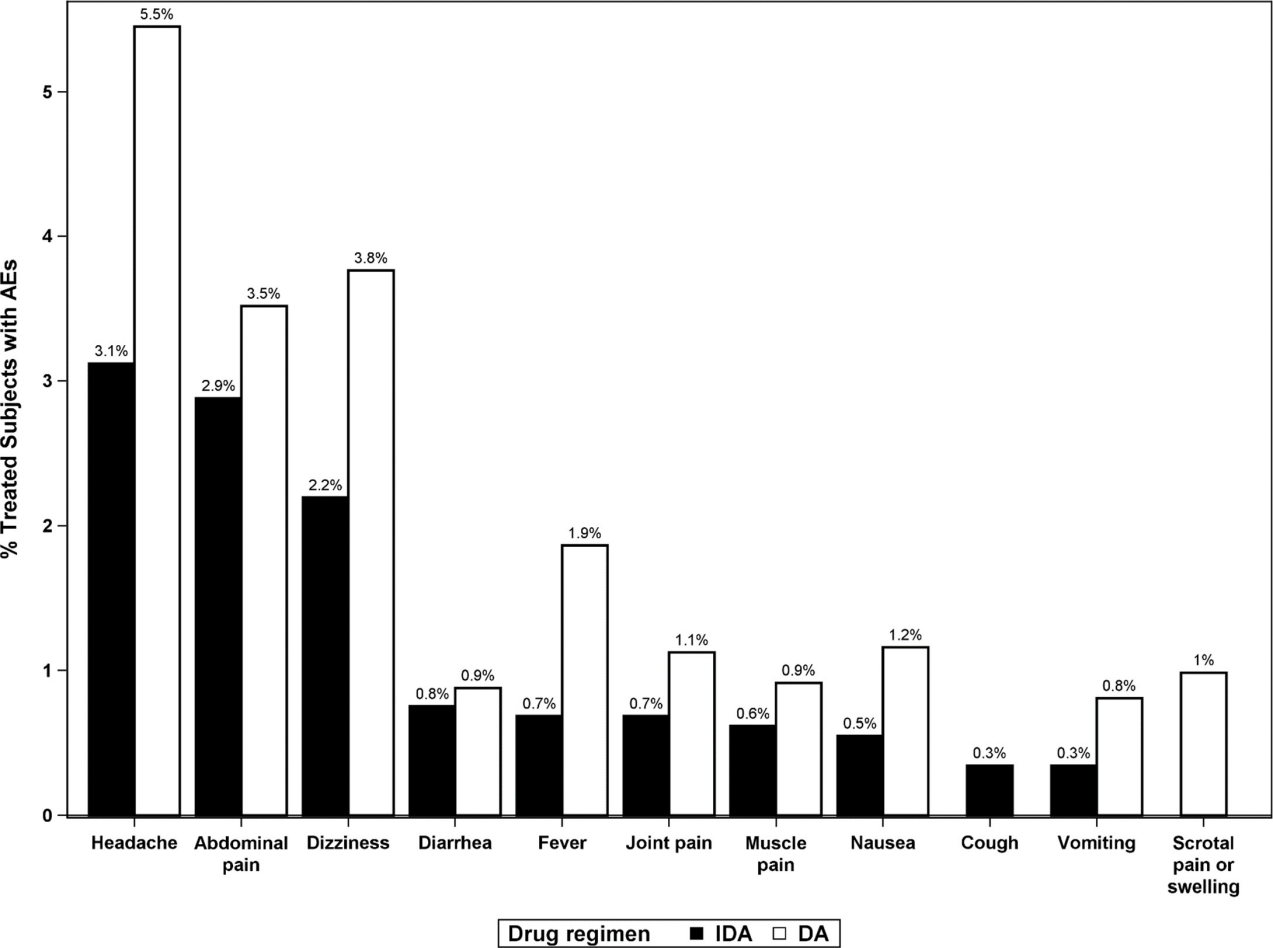
Frequencies of the most commonly observed adverse events (AEs) by type of treatment. Frequencies are expressed as percentages of participants who were assessed for AEs after treatment.

A total of 1,187 AEs were reported by 812 study participants who reported at least one AE. The most frequent AEs reported in both treatment areas were headache, abdominal pain and dizziness. The types and proportions of AEs observed and reported among participants were similar after DA and IDA treatment (Fig 6). Scrotal (pain or swelling) AEs were reported by 1.3% (35/2,664) of men. They were more commonly reported after DA (2.1%, 28/1315) than after IDA (0.5%, 7/1,349) (*P* = 0.0003) and among Mf positive men (8.6%, 7/81) than among Mf negative men (1.1%, 28/2549) (*P<0*.*0001*). Most scrotal AEs were of severity grade 1 (32/35, 91.4%). After DA, two men reported grade 2 scrotal AEs and one man reported a grade 3 scrotal AE. All scrotal AEs (7) reported in the IDA treatment group were grade 1.

### Efficacy study results

In January 2018, 83.9% (489/583) of participants who were CFA positive at baseline were retested for the efficacy study. This included 94 participants who were microfilaremic at baseline. The follow up rates were similar for participants who had been treated with IDA and DA (Fig 2).

**Fig 7:**
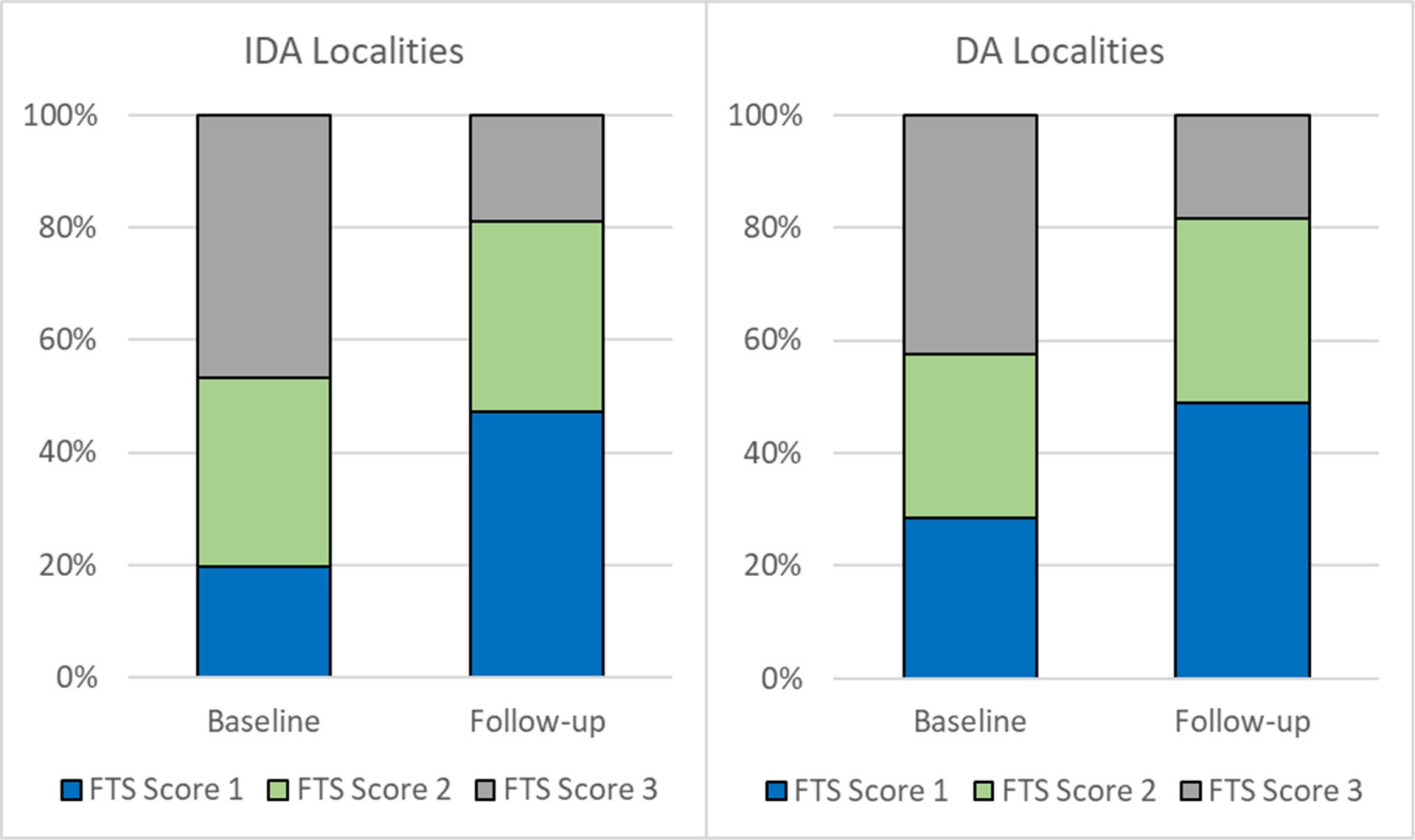
FTS score distribution at baseline and one year after treatment by type of treatment. FTS, Filariasis Test Strip.

The proportions of participants who were CFA positive at baseline and who became CFA negative after one year were similar in those who received IDA (20.5%, 41/200) and DA (25.6%, 74/289) (*P* = 0.3). The largest proportion of CFA-positive participants received an FTS score of 3 at baseline, however most participants with persistent CFA had an FTS score of 1 one year after treatment (Fig 7).The proportion of FTS score 1 changed from 19.7% to 47.2% in the IDA arm (*P*<0.001) and from 28.5% to 48.9% in the DA arm (*P*<0.001). When comparing treatment group to each other, there was no statistically significant difference in the distribution of FTS scores between groups at visit 1 (*P* = 0.5) or visit 2 (*P* = 0.9).

**Fig 8:**
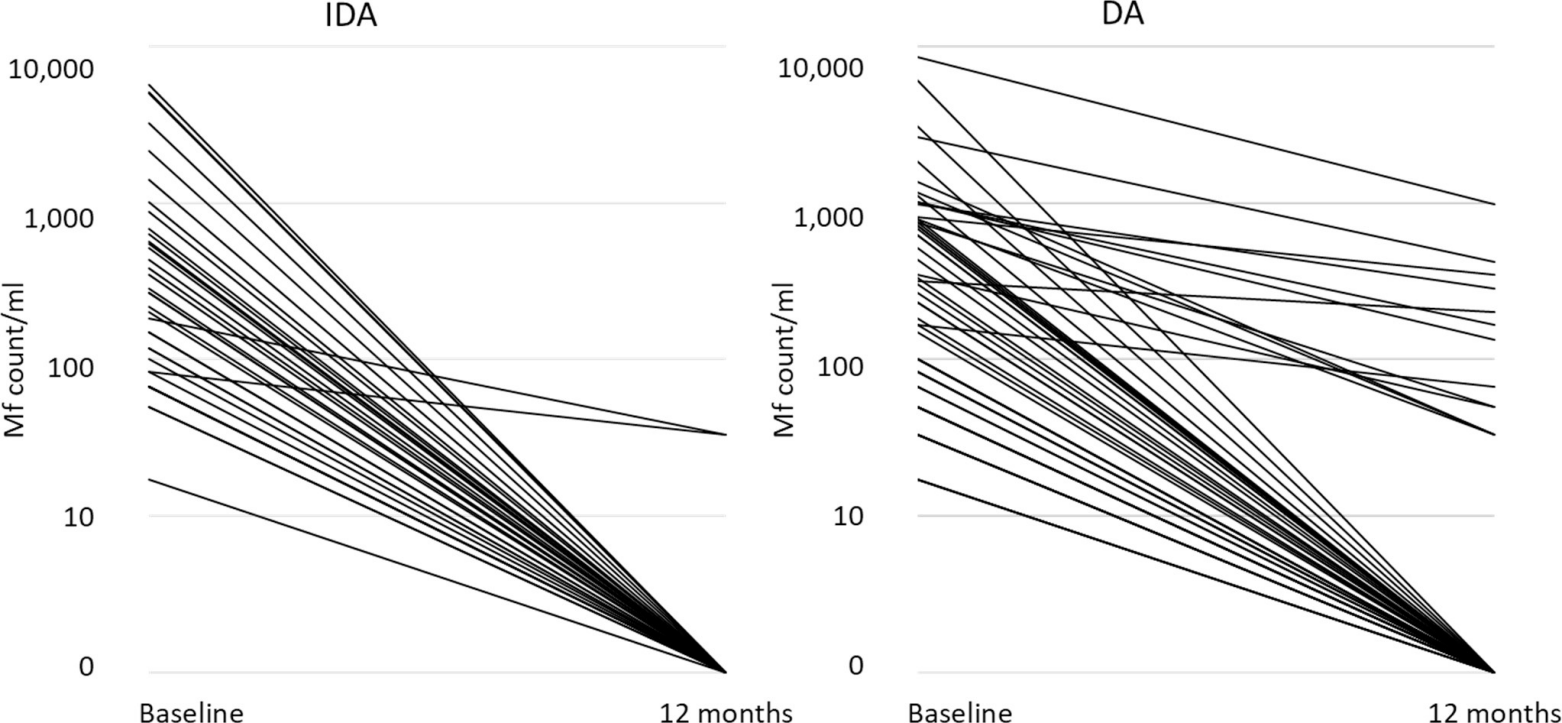
Microfilaremia reduction 12 months after treatment by type of treatment. DA, diethylcarbamazine plus albendazole; IDA, ivermectin plus diethylcarbamazine and albendazole; Mf, microfilariae.

One year after treatment, significantly more participants who were Mf positive at baseline became Mf negative after IDA (94.4%, 34/36) compared to after DA (75.9%, 44/58) (*P* = 0.02). The two participants who remained Mf positive after IDA had Mf counts of 3,050 Mf/mL and 1383 Mf/mL at baseline. One year after treatment, the Mf counts in both participants were 33/mL (Fig 8). Two participants who were CFA positive and Mf negative at baseline were Mf positive one year after DA. The FTS score for both participants was 2 at baseline. One year after treatment, the FTS score of the first participant was 3 and the Mf count was 6,667 Mf/mL; the FTS score of the second participant was 1 and the Mf count was 50Mf/mL. Overall, the Mf geometric mean one year after treatment among Mf positive participants at baseline decreased from 250 Mf/mL to 124 Mf/mL (*P* = 0.07). The decrease was not significant in the DA arm (223 Mf/mL vs. 148 Mf/mL, *P* = 0.3) whereas it was in the IDA arm (305 Mf/mL vs. 33 Mf/mL, *P* = 0.04).

Gender was not associated with total clearance of Mf or CFA after either treatment using chi-squared tests. Younger age was associated with conversion to FTS negativity in participants after DA but not after IDA. Age was not associated with conversion to Mf negativity (Table 5).

**Table 5:** Proportion of CFA and Mf positive participants with negative conversion 12-months after treatment by type of treatment, sex, and age.

	IDA				DA			
	CFA (+) to CFA (-) n (%)	P value*	Mf (+) to Mf (-) n (%)	P value*	CFA (+) to CFA (-) n (%)	P value*	Mf (+) to Mf (-) n (%)	P value*
Female	20/86 (23.4)	0.4	10/10 (100.0)	0.4	31/105 (29.5)	0.2	8/12 (66.7)	0.4
Male	21/114 (18.4)		24/26 (92.3)		43/184 (23.4)		36/46 (78.3)	
Age (years)								
5–9	5/25 (20.0)	0.8	6/6 (100.0)	0.2	15/28 (53.6)	0.001	0/0 (0.0)	0.9
10–17	5/30 (16.7)		3/4 (75.0)		9/27 (33.3)		3/4 (75.0)	
18 +	31/145 (21.4)		25/26 (96.2)		50/234 (21.4)		41/54 (75.9)	

*Chi squared P value

DA, diethylcarbamazine plus albendazole; IDA, ivermectin plus diethylcarbamazine and albendazole; CFA, circulating filarial antigenemia; Mf, microfilariae

## Discussion

This cluster-randomized, open-label community study represents the first evaluation of the safety and efficacy of IDA MDA for LF in Haiti. IDA was well tolerated by the study participants and was more effective for clearing Mf than DA. Both treatments reduced CFA levels, but relatively few participants completely cleared CFA after a single dose of either DA or IDA.

The Haiti study was part of a multicenter safety study in five countries that found that IDA was well tolerated in LF-endemic communities [15]. Haiti was the only country where the proportion of participants with AE was significantly lower in people taking the IDA regimen than in people taking the DA regimen. Although we do not know with certainty the reason for this, several factors may have contributed. Localities assigned to receive DA were treated during a rainy season characterized by heavy floods and long period of rains. This may have led to increases in vector and non-vector borne infections that were counted as AEs during that period. Another possible explanation for fewer AEs after IDA might be that the ancillary benefits provided by IVM such as improvement of scabies [21] and strongyloidiasis [22]. These benefits may have decreased the number of AEs reported by participants who received IDA compared to those who received DA, because IDA recipients may have experienced an improvement of symptoms related to these two infections. Additionally, despite random assignment of localities to the two drug regimens, the proportion of participants who were Mf or CFA positive at baseline were higher in DA treatment areas than in IDA treatment areas. However, AE rates in Mf positive participants were not significantly different in the DA and IDA treatment areas.

Importantly, as was observed in other studies, most AEs reported by participants receiving IDA were mild. Only three SAE were recorded during the study, all after DA. No SAEs were recorded after IDA, which gave us confidence that the SAE rate for this study was less than 0.1% among participants treated with IDA. These AEs were categorized as serious because participants were admitted to the hospital late in the day and stayed overnight for observation; all three patients were discharged the next day. While they satisfied the criteria for SAE, the severity of these events was relatively low (grade 3), and AE durations were short. Regardless of drug regimen, the most common AEs reported were headache, dizziness and abdominal pain, which was similar to those reported by participants in an early study of IDA for LF [7]. These same AEs were also commonly reported among participants from other countries in the multicenter study [15].

In our study, where the overall Mf rate at baseline was 1.9%, we found that microfilaremic participants were more likely to report AEs (37.4%) than amicrofilaremic participants (13.7%). As opposed to other countries included in the multicenter study, the proportion of microfilaremic patients who experienced at least one AE did not differ significantly between those who took DA and IDA regimens (Table 4). Previous reports on AEs following single dose treatment of LF have also showed that the AE rates following DEC or IVM treatment were typically higher in microfilaremic participants [23]. One safety study carried out in the commune of Leogane in Haiti after MDA with DEC and ALB reported that in a population where the Mf prevalence ranged from 1% to 16%, 24% (17,421/71,187) of the population treated during MDA reported AE [24].

Most previous studies assessing the safety of antifilarial drugs have been carried out in persons with microfilaremia. For example, studies in Papua New Guinea and Ivory Coast that assessed AEs in persons with high level microfilaremia reported AEs rate of 83% and 100% respectively among participants who received IDA [7, 8]. A randomized placebo-controlled study of microfilaremic children in Haiti who took ALB and IVM alone or in combination found that the most commonly reported AE were fever, headache, myalgias and cough. These AEs were recorded more frequently for children who had taken IVM alone or in combination with ALB. Symptom severity was associated with higher Mf counts [25]. Another study in Haiti compared DEC and IVM for treatment of bancroftian filariasis in microfilaremic adults. Ninety percent of participants receiving an initial dose (1 mg) of IVM reported at least one AE, most commonly headache, fever and myalgias. Only participants who received DEC developed scrotal reactions indicative of death of adult worms [26].

As was observed in other countries participating in this multicenter study, women were more likely than men and adults were more likely than children to report AEs after adjusting for infection status. This finding suggests that the observed differences in AE rates in these two groups are not attributable to differences in infection rates. A review of AE following single dose treatment of LF reported that of those studies that reported gender-specific AE rates, most did not find significant differences in AE rates between genders [23]. In Haiti, one study reported more moderate AEs among men than women following MDA with ALB and DEC [24]. In that study, 1.5% of males reported localized scrotal reactions severe enough to interfere with daily activities. This accounted for 40% of all moderate AEs recorded during the study. In our study, scrotal pain or swelling was reported by 2.1% of men after IDA and by 0.5% of men after DA.

One limitation of this study is that less than half of the population in the initial census agreed to participate in the study. Considerable effort was undertaken to increase participation. For example, study teams enrolled participants in the afternoons and evenings to increase the chance of finding people at home after school or work, community leaders repeated announcements throughout the study and study teams carried out multiple mop up visits in each locality. Variable enrollment rates by community illustrate how acceptability of a public health intervention can vary locally. We noted that the low participation rate in the study was not related to high baseline infection rates. For example, the locality of Balan had one of the lowest participation rates (40%) but the CFA and Mf prevalence were the lowest before treatment, 4.6% and 0.4% respectively. Complacency due to many prior rounds of MDA for LF in this area may have contributed to the low participation rate. Also, proximity to a large town may have played a role, as many studies have documented higher compliance in rural settings compared to cities [27–29]. LF elimination programs will need to develop social mobilization and drug distribution strategies specifically tailored to reach non-adherent populations. This is particularly relevant for the LF elimination program in Haiti. Transmission assessment surveys (TAS) or sentinel site surveys have documented LF prevalence above the targets that are required for stopping MDA after 10 years of MDA in several communes that had high baseline prevalence. The reasons for this situation are probably multi-factorial (ecological, programmatic, socio-cultural, epidemiologic). In general, ensuring high MDA coverage in all persons with LF infection will be critical to elimination regardless of drug regimen used.

Overall, CFA and Mf prevalence in the study areas (IDA and DA) just prior to MDA were 9.7% and 1.9% respectively. Despite the study area being small and localities bordering each other, infection rates were significantly different in the localities studied. CFA was present in 5.3% of children 5–9 years old; 8 of the CFA positive children were also Mf positive. The baseline results among children (many of whom were born after years of MDA in the study area) provide strong evidence for ongoing LF transmission in these communities. This is further supported by the proportions of adults 20 years old and older who were positive for CFA (15.0%) and Mf (3.3%). About 45% of the participants who were CFA-positive at baseline had an FTS score of three; Mf prevalence was higher in persons with high FTS scores. This relationship has been observed in studies conducted in four African countries in areas that had not previously received MDA [17].

Baseline infection prevalence before MDA and MDA coverage results are reported by commune in Haiti and not by localities. In the commune of Quartier Morin, the infection rate in 2001 was reported to be 39% (CFA among 6–11 years old children) [30] and reported coverages during 2012–2015 were consistently high, around 100%. Coverage surveys (results not available) have been recently undertaken by the MSPP to validate these results. One wonders whether the variability in infection prevalence at baseline in this study reflects focality in parameters such as original pre-MDA infection rates, mosquito factors, or the cumulative effects of variable MDA compliance and systematic non-adherence related to local factors [31]. A gender difference in CFA and Mf prevalence at baseline was also observed. This could be related to differences in exposure to mosquito bites or to lower MDA adherence over years. Howerver, we observed than fewer older people (who were more likely to have been exposed to LF) participated in our study but that men and women’s participation was relatively evenly distributed among different age groups (Fig 4). It is also possible that age-based dosing of DEC in the national program systematically led to under dosing of males relative to females [32].

More than 80% of the CFA positive participants at baseline were retested one year after treatment. The change in antigenemia did not differ between arms and about three quarters of all participants remained CFA positive after one year. However, FTS scores tended to decrease after treatment. Almost 95% of the participants who were Mf positive at baseline were amicrofilaremic one year after receiving IDA compared to 74.9% for participants who received DA. These results were similar to results found in the clinical trials in Ivory Coast and Papua New Guinea and confirmed that the 3-drug therapy has dramatic microfilaricidal and sterilizing effect with a partial macrofilaricidal effect [7, 8]. Our results confirm that using CFA to assess endpoints for MDA after IDA will be challenging. TAS routinely implemented to determine if MDA can be stopped are implemented in children 6–7 years old using FTS. In our study, we looked at changes in CFA and Mf prevalence after IDA in children 5–9 years old. In that age-group, all children became Mf negative one year after treatment, but 80% remained antigenemic. Persistence of antigenemia was also seen in older people (Table 5). These results suggest that using the TAS methodology to measure the true impact of the 3-drug regimen compared to the 2-drug regimen in places that have implemented IDA MDA for one or two years as recommended by WHO [33] might be challenging since the impact of these two regimens on antigenemia is similar.

Based in part on results from clinical trials and the multicenter safety study [15], WHO published a guideline in 2017 for alternative MDA regimens to eliminate LF. One special setting in which WHO recommends the use of annual IDA rather than annual DA is in implementation units (communes in the case of Haiti) that have not met the epidemiological thresholds in sentinel and spot-check site survey or in TAS despite meeting drug coverage targets [34]. This is the case for most communes in Haiti where MDAs are still needed. The efficacy results from this study suggest that substitution of IDA for DA may accelerate LF elimination in Haiti. However, strengthening operational aspects of MDA to ensure high community compliance (including in previously non-adherent groups) and operational research to determine the best way to measure the impact of MDAs and endpoints for MDA programs that use IDA are needed.

## References

[1] Utzinger J, Becker SL, Knopp S, Blum J, Neumayr AL, Keiser J, et al Neglected tropical diseases: diagnosis, clinical management, treatment and control. Swiss medical weekly. 2012;142:w13727 Epub 2012/11/28. 10.4414/smw.2012.13727 . 23180107

[2] World Health Organization (1997). Elimination of lymphatic filariasis as a public health problem—resolution of the executive board of the WHO. Available from: http://www.who.int/neglected_diseases/mediacentre/WHA_50.29_Eng.pdf

[3] World Health Organisation (2018). Global Programme to Eliminate Lymphatic Filariasis. Available from: http://www.who.int/lymphatic_filariasis/elimination-programme/en/.

[4] Transmission assessment surveys in the Global Programme to Eliminate Lymphatic Filariasis: WHO position statement. Releve epidemiologique hebdomadaire / Section d'hygiene du Secretariat de la Societe des Nations = Weekly epidemiological record / Health Section of the Secretariat of the League of Nations. 2012;87(48):478–82. Epub 2012/12/12. . 23213667

[5] World Health Organization. Global programme to eliminate lymphatic filariasis: progress report, 2018. Releve epidemiologique hebdomadaire / Section d'hygiene du Secretariat de la Societe des Nations = Weekly epidemiological record / Health Section of the Secretariat of the League of Nations. 2019;94(41):457–72. Epub 2019/10/11.

[6] King CL, Suamani J, Sanuku N, Cheng YC, Satofan S, Mancuso B, et al A Trial of a Triple-Drug Treatment for Lymphatic Filariasis. The New England journal of medicine. 2018;379(19):1801–10. Epub 2018/11/08. 10.1056/NEJMoa1706854 30403937 PMC6194477

[7] Thomsen EK, Sanuku N, Baea M, Satofan S, Maki E, Lombore B, et al Efficacy, Safety, and Pharmacokinetics of Coadministered Diethylcarbamazine, Albendazole, and Ivermectin for Treatment of Bancroftian Filariasis. Clinical infectious diseases: an official publication of the Infectious Diseases Society of America. 2015 Epub 2015/10/22. 10.1093/cid/civ882 . 26486704

[8] Edi C, Bjerum CM, Ouattara AF, Chhonker YS, Penali LK, Meite A, et al Pharmacokinetics, safety, and efficacy of a single co-administered dose of diethylcarbamazine, albendazole and ivermectin in adults with and without Wuchereria bancrofti infection in Cote d'Ivoire. PLoS neglected tropical diseases. 2019;13(5):e0007325 Epub 2019/05/21. 10.1371/journal.pntd.0007325 31107869 PMC6550417

[9] Irvine MA, Stolk WA, Smith ME, Subramanian S, Singh BK, Weil GJ, et al Effectiveness of a triple-drug regimen for global elimination of lymphatic filariasis: a modelling study. The Lancet Infectious diseases. 2017;17(4):451–8. Epub 2016/12/26. 10.1016/S1473-3099(16)30467-4 . 28012943

[10] World Health Organisation (2006). The safety of medicines in public health programmes: pharmacovigilance an essential tool. Available from: http://www.who.int/medicines/areas/quality_safety/safety_efficacy/Pharmacovigilance_B.pdf.

[11] Layton D, Hazell L, Shakir SA. Modified prescription-event monitoring studies: a tool for pharmacovigilance and risk management. Drug safety. 2011;34(12):e1–9. Epub 2011/11/15. 10.2165/11593830-000000000-00000 . 22077508

[12] de Rochars MB, Kanjilal S, Direny AN, Radday J, Lafontant JG, Mathieu E, et al The Leogane, Haiti demonstration project: decreased microfilaremia and program costs after three years of mass drug administration. The American journal of tropical medicine and hygiene. 2005;73(5):888–94. Epub 2005/11/12. . 16282299

[13] Oscar R, Lemoine JF, Direny AN, Desir L, Beau de Rochars VE, Poirier MJ, et al Haiti National Program for the elimination of lymphatic filariasis—a model of success in the face of adversity. PLoS neglected tropical diseases. 2014;8(7):e2915 Epub 2014/07/18. 10.1371/journal.pntd.0002915 25032697 PMC4102456

[14] Lammie PJ, Eberhard ML, Addiss DG, Won KY, Beau de Rochars M, Direny AN, et al Translating Research into Reality: Elimination of Lymphatic Filariasis from Haiti. The American journal of tropical medicine and hygiene. 2017;97(4_Suppl):71–5. Epub 2017/10/25. 10.4269/ajtmh.16-0669 29064364 PMC5676631

[15] Weil GJ, Bogus J, Christian M, Dubray C, Djuardi Y, Fischer PU, et al The safety of double- and triple-drug community mass drug administration for lymphatic filariasis: A multicenter, open-label, cluster-randomized study. PLoS medicine. 2019;16(6):e1002839 Epub 2019/06/25. 10.1371/journal.pmed.1002839 31233507 PMC6590784

[16] Chesnais CB, Missamou F, Pion SD, Bopda J, Louya F, Majewski AC, et al Semi-quantitative scoring of an immunochromatographic test for circulating filarial antigen. The American journal of tropical medicine and hygiene. 2013;89(5):916–8. Epub 2013/09/11. 10.4269/ajtmh.13-0245 24019435 PMC3820335

[17] Chesnais CB, Awaca-Uvon NP, Bolay FK, Boussinesq M, Fischer PU, Gankpala L, et al A multi-center field study of two point-of-care tests for circulating Wuchereria bancrofti antigenemia in Africa. PLoS neglected tropical diseases. 2017;11(9):e0005703 Epub 2017/09/12. 10.1371/journal.pntd.0005703 28892473 PMC5608416

[18] National Cancer Institute (2017) NCI Common Terminology Criteria for Adverse Events (CTCAE) v.5.0. Available at https://ctep.cancer.gov/protocolDevelopment/electronic_applications/docs/CTCAE_v5_Quick_Reference_5x7.pdf. Accessed 04 June 2018.

[19] Gaddale JR. Clinical Data Acquisition Standards Harmonization importance and benefits in clinical data management. Perspectives in clinical research. 2015;6(4):179–83. Epub 2015/12/02. 10.4103/2229-3485.167101 26623387 PMC4640009

[20] Medical Dictionary for Regulatory Activities [cited 2017 Feb 3]. Available from: https://www.meddra.org/.

[21] Thomas C, Coates SJ, Engelman D, Chosidow O, Chang AY. Part I—Ectoparasites: Scabies. Journal of the American Academy of Dermatology. 2019 Epub 2019/07/17. 10.1016/j.jaad.2019.05.109 . 31310840

[22] Henriquez-Camacho C, Gotuzzo E, Echevarria J, White AC Jr., Terashima A, Samalvides F, et al Ivermectin versus albendazole or thiabendazole for Strongyloides stercoralis infection. Cochrane database of systematic reviews (Online). 2016;(1):Cd007745 Epub 2016/01/19. 10.1002/14651858.CD007745.pub3 26778150 PMC4916931

[23] Budge PJ, Herbert C, Andersen B, Weil GJ. Adverse events following single dose treatment of lymphatic filariasis: Observations from a review of the literature. PLoS neglected tropical diseases. 2018;12(5):e0006454 Epub 2018/05/17. 10.1371/journal.pntd.0006454 . 29768412 PMC5973625

[24] McLaughlin SI, Radday J, Michel MC, Addiss DG, Beach MJ, Lammie PJ, et al Frequency, severity, and costs of adverse reactions following mass treatment for lymphatic filariasis using diethylcarbamazine and albendazole in Leogane, Haiti, 2000. The American journal of tropical medicine and hygiene. 2003;68(5):568–73. Epub 2003/06/19. 10.4269/ajtmh.2003.68.568 . 12812348

[25] Addiss DG, Beach MJ, Streit TG, Lutwick S, LeConte FH, Lafontant JG, et al Randomised placebo-controlled comparison of ivermectin and albendazole alone and in combination for Wuchereria bancrofti microfilaraemia in Haitian children. Lancet. 1997;350(9076):480–4. Epub 1997/08/16. 10.1016/S0140-6736(97)02231-9 . 9274584

[26] Richards FO Jr., Eberhard ML, Bryan RT, McNeeley DF, Lammie PJ, McNeeley MB, et al Comparison of high dose ivermectin and diethylcarbamazine for activity against bancroftian filariasis in Haiti. The American journal of tropical medicine and hygiene. 1991;44(1):3–10. Epub 1991/01/11. 10.4269/ajtmh.1991.44.3 . 1996738

[27] Silumbwe A, Zulu JM, Halwindi H, Jacobs C, Zgambo J, Dambe R, et al A systematic review of factors that shape implementation of mass drug administration for lymphatic filariasis in sub-Saharan Africa. BMC public health. 2017;17(1):484 Epub 2017/05/24. 10.1186/s12889-017-4414-5 28532397 PMC5441010

[28] Krentel A, Fischer PU, Weil GJ. A review of factors that influence individual compliance with mass drug administration for elimination of lymphatic filariasis. PLoS neglected tropical diseases. 2013;7(11):e2447 Epub 2013/11/28. 10.1371/journal.pntd.0002447 24278486 PMC3836848

[29] Adams AM, Vuckovic M, Birch E, Brant TA, Bialek S, Yoon D, et al Eliminating Neglected Tropical Diseases in Urban Areas: A Review of Challenges, Strategies and Research Directions for Successful Mass Drug Administration. Tropical medicine and infectious disease. 2018;3(4). Epub 2018/11/25. 10.3390/tropicalmed3040122 30469342 PMC6306919

[30] Drexler N, Washington CH, Lovegrove M, Grady C, Milord MD, Streit T, et al Secondary mapping of lymphatic filariasis in Haiti-definition of transmission foci in low-prevalence settings. PLoS neglected tropical diseases. 2012;6(10):e1807 Epub 2012/10/17. 10.1371/journal.pntd.0001807 23071849 PMC3469481

[31] Hollingsworth TD, Adams ER, Anderson RM, Atkins K, Bartsch S, Basanez MG, et al Quantitative analyses and modelling to support achievement of the 2020 goals for nine neglected tropical diseases. Parasites & vectors. 2015;8:630 Epub 2015/12/15. 10.1186/s13071-015-1235-1 26652272 PMC4674954

[32] Goss CW, O'Brian K, Dubray C, Fischer PU, Hardy M, Jambulingam P, et al Dosing pole recommendations for lymphatic filariasis elimination: A height-weight quantile regression modeling approach. PLoS neglected tropical diseases. 2019;13(7):e0007541 Epub 2019/07/18. 10.1371/journal.pntd.0007541 31314753 PMC6663033

[33] WHO Guidelines Approved by the Guidelines Review Committee. Guideline: Alternative Mass Drug Administration Regimens to Eliminate Lymphatic Filariasis. Geneva: World Health Organization Copyright (c) World Health Organization 2017; 2017. 29565523

[34] Summary of global update on preventive chemotherapy implementation in 2016: crossing the billion. Releve epidemiologique hebdomadaire / Section d'hygiene du Secretariat de la Societe des Nations = Weekly epidemiological record / Health Section of the Secretariat of the League of Nations. 2017;92(40):589–93. Epub 2017/10/07. . 28984120

